# Genetic Diversity, Heteroplasmy, and Recombination in Mitochondrial Genomes of *Daphnia pulex*, *Daphnia pulicaria*, and *Daphnia obtusa*

**DOI:** 10.1093/molbev/msac059

**Published:** 2022-03-24

**Authors:** Zhiqiang Ye, Chaoxian Zhao, R. Taylor Raborn, Man Lin, Wen Wei, Yue Hao, Michael Lynch

**Affiliations:** Center for Mechanisms of Evolution, Biodesign Institute, Arizona State University, Tempe, AZ 85287, USA

**Keywords:** *Daphnia*, heteroplasmy, hybridization, mitochondria, nucleotide diversity, purifying selection

## Abstract

Genetic variants of mitochondrial DNA at the individual (heteroplasmy) and population (polymorphism) levels provide insight into their roles in multiple cellular and evolutionary processes. However, owing to the paucity of genome-wide data at the within-individual and population levels, the broad patterns of these two forms of variation remain poorly understood. Here, we analyze 1,804 complete mitochondrial genome sequences from *Daphnia pulex*, *Daphnia pulicaria*, and *Daphnia obtusa*. Extensive heteroplasmy is observed in *D. obtusa*, where the high level of intraclonal divergence must have resulted from a biparental-inheritance event, and recombination in the mitochondrial genome is apparent, although perhaps not widespread. Global samples of *D. pulex* reveal remarkably low mitochondrial effective population sizes, <3% of those for the nuclear genome. In addition, levels of population diversity in mitochondrial and nuclear genomes are uncorrelated across populations, suggesting an idiosyncratic evolutionary history of mitochondria in *D. pulex*. These population-genetic features appear to be a consequence of background selection associated with highly deleterious mutations arising in the strongly linked mitochondrial genome, which is consistent with polymorphism and divergence data suggesting a predominance of strong purifying selection. Nonetheless, the fixation of mildly deleterious mutations in the mitochondrial genome also appears to be driving positive selection on genes encoded in the nuclear genome whose products are deployed in the mitochondrion.

## Introduction

Unlike the nuclear genome, mitochondrial genomes are generally inherited maternally, have hundreds to thousands of copies present in each cell (Sato and Sato 2013), and have very little recombination in animals (Berlin et al. 2004; Hagström et al. 2014). Because of their unique evolutionary history and ease of sequencing, mitochondrial genomes have been widely applied in studies of population structure, demographic history, and relationships among populations and/or species. For example, numerous investigations into mtDNA variation at the population level have revealed substantial between-individual nucleotide diversity in animal species (Bazin et al. 2006; Nabholz et al. 2008, 2009; Allio et al. 2017; Mackintosh et al. 2019; James and Eyre-Walker 2020). Less is known about the actual mechanisms driving such variation, although large-scale whole-genome sequencing in humans has been revealing selection against certain mutations (Wei et al. 2017, 2019).

Although molecular variation is shaped by selection, mutation, recombination, gene flow, and random genetic drift, it has been argued that there is no correlation between mtDNA genetic diversity and effective population size (*N*_e_) in animals (Bazin et al. 2006). However, to imply *N*_e_, such analyses rely on the questionable assumption of an invariant mitochondrial mutation rate. For nuclear genes, there is an inverse relationship between *N*_e_ and the mutation rate, associated with a 1,000-fold range of variation in the latter, the net result being only slight variation in molecular diversity across orders of magnitude differences in *N*_e_ (Lynch et al. 2016). As significant mutation-rate variation exists for organelle genomes, even within metazoans (Santos et al. 2005; Haag-Liautard et al. 2008; Denver et al. 2009; Krasovec et al. 2019; Ho et al. 2020), incorrect inferences on population size will result when assuming diversity measures to be proportional to *N*_e_. Given additional uncertainties on the degree to which purifying versus positive selection defines patterns of mtDNA genetic diversity and divergence (Nabholz et al. 2008, 2009; Allio et al. 2017; James et al. 2017), there is considerable room for refined studies of mitochondrial evolution. The necessary data are now readily accessible, as population-genomic sequencing projects focused on nuclear genomes generally generate as a side-product organelle-genome sequences with coverages well above 100×.

Unlike the situation in nuclear genomes, where each diploid individual acquires a haploid genomic complement from each parent, organelle genomes are thought to be uniparentally (usually maternally) inherited in most animals, leading to the expectation of a high degree of homoplasmy within individuals. Although variation initiated by mutation at the within-individual level must lead to some level of heteroplasmy (a mixture of mtDNA haplotypes in single cells or among cells within individuals), earlier studies based on bulk Sanger sequencing had little power to identify rare variants. The high depths of coverage yielded by next-generation sequencing technologies now allow confident identification of heteroplasmic mutations with frequencies as low as 1%, leading to the suggestion that mitochondrial heteroplasmy is common in humans (Wallace and Chalkia 2013; Ye et al. 2014; Li et al. 2015; Stewart and Chinnery 2015), and by extension likely so in other organisms.

Heteroplasmy may also arise via rare cases of biparental inheritance. Even with mitochondrial genome inheritance being primarily maternal, hundreds to thousands of mitochondria may be present in each metazoan sperm cell (Sato and Sato 2013), so the possibility of low-level paternal leakage cannot be entirely ruled out. This then raises further questions as to whether mitochondrial genomes are actually propagated in an effectively clonal fashion, as is often assumed. Organelle genomes are physically capable of recombination (Thyagarajan et al. 1996; Kazak et al. 2012), but the opportunities for generating novel recombinant genotypes are restricted, as this requires the participation of two molecules differing at a minimum of two nucleotide sites, an unlikely mutational scenario with rapid within-individual sorting. Thus, whereas mitochondrial genomes may undergo recombination in some plant and fungal species (Anderson et al. 2001; Shedge et al. 2007; Fritsch et al. 2014; Gualberto and Newton 2017), it is generally thought that recombination is negligible in animals, at least in terms of generating novel allelic combinations (Berlin et al. 2004; Piganeau et al. 2004; Hagström et al. 2014).

Large-scale studies at the population-genomic level in the aquatic microcrustacean *Daphnia* provide an opportunity to explore all of these issues in further depth. *Daphnia* are model organisms used extensively in ecological, physiological, and ecotoxicological studies. However, while much is known about the nuclear genetic diversity of this species at the population level (e.g., Lynch et al. 2017; Maruki et al. 2022), and fully assembled mitochondrial genomes have been available for some time (Crease 1999; Geng et al. 2016), we lack an understanding of the extent of population-genetic and phylogenetic features of mitochondrial variation and the underlying determinants of such variation. Using whole-genomic sequencing data from 1,804 clones, we now fill this gap by investigating mitochondrial nucleotide diversity at the within-individual, between-individual, between-population, and between-species levels in *D. pulex*, *D. pulicaria*, and *D. obtusa*.

The *D. pulex* complex consists of a series of morphologically similar taxa, with the potential to produce viable hybrid progeny in some cases (Agar 1920; Heier and Dudycha 2009), although there are uncertainties regarding the long-term significance of introgression events. For example, hybridization between the two primary species, *D. pulex* and *D. pulicaria*, can result in discordant phylogenetic trees based on mitochondrial versus nuclear genes (Marková et al. 2013; Ye et al. 2021), although the majority of such hybridization events seem to result in the origin of obligately parthenogenetic offspring and the cessation of further gene flow (Xu et al. 2015). In addition, based on sequences of the mitochondrial cytochrome c oxidase subunit 1 gene, Penton et al. (2004) suggested that North American (NA) *D. obtusa* consists of two morphologically cryptic species, with evidence of potential ongoing hybridization. Mito-nuclear discordance has been found in other *Daphnia* species complexes (e.g., *D. galeata*, *D. longispina*, and *D. cucullata*), suggesting historical differences in maternal versus paternal gene flow (Thielsch et al. 2017).

The current study encompasses *D. pulex*, *D. pulicaria*, and *D. obtusa*, with isolates of the first species derived from three continents. Although *D. obtusa* is quite distinct from *D. pulex/pulicaria*, there are nomenclatural issues with respect to the latter, as NA *D. pulex* and *D. pulicaria* appear to be much more closely related to each other than to European *D. pulex/pulicaria*, and there are even some uncertainties regarding the distinctiveness of *D. pulex* and *D. pulicaria* within continents (Pfrender et al. 2000; Vergilino et al. 2011; Ma et al. 2019). These issues will be clarified further in the following analyses.

Using phylogenetic and population-genetic analyses, we reconstruct the relationships of complete mitochondrial haplotypes from *D. pulex*, *D. pulicaria*, and *D. obtusa* across the northern hemisphere, and evaluate the degree to which polymorphic variants distribute within and among populations. Parallel data for whole-nuclear genomes from 10 *D. pulex* populations (Maruki et al. 2022) allow for an unprecedented level of comparison of the degree of congruence between genetic diversity at the mitochondrial and nuclear DNA levels. We also evaluate the extent to which *Daphnia* mitochondrial genes experience purifying selection, and the consequences of rapid mitochondrial gene evolution for the coevolution of nuclear genes with interacting products. Use of the known mutation rates in both the mitochondrial and nuclear genomes of *D. pulex* leads to the conclusion that the effective population size of the former is <3% of the latter, and that this is likely a consequence of strong purifying selection operating on the largely nonrecombining organelle genome. This work represents one of the most comprehensive studies to date of patterns of mitochondrial variation in any species.

## Materials and Methods

### Sample Preparation and Sequencing

*Daphnia pulex* were collected from North America, Europe (Czech Republic), and Asia (China), whereas all *D. pulicaria* and *D. obtusa* isolates were derived from North America (for more details see supplementary table S1, Supplementary Material online). Individual isolates, DNA extraction, and genome sequencing were performed as described in Maruki et al. (2022). To maximize the likelihood that each individual would originate from a unique genotype, we collected hatchlings in the early spring before the occurrence of subsequent reproduction. Individual isolates were clonally maintained in the laboratory for three generations, and DNA was extracted from 96 isolates per population. The library for each sample was prepared using a Bioo/Nextera kit, followed by tagging with unique oligomer barcodes. Samples from the same population were pooled for sequencing using the Illumina NextSeq 500 or HighSeq 2500 platform, 100 or 150 bp paired-end short reads were generated for each sample. We also included 32 obligately asexual clones sequenced by Tucker et al. (2013) and Xu et al. (2015) into our analysis.

### Mitochondrial Genome Assembly

Due to high genetic diversity among *Daphnia* clades in our study, we used specific mitochondrial reference genomes for each of the five clades (NA *D. pulex* and *D. pulicaria*; European *D. pulex*; Asian *D. pulex*; *D. obtusa* clade I; and *D. obtusa* clade II) in our analysis. Because of the similarity between the mitochondrial genomes of NA *D. pulex* and *D. pulicaria*, we used *D. pulex* GenBank accession number AF117817.1 as the reference mitochondrial genome for the NA clade. Sequence from GenBank accession number KT003819.1 (Geng et al. 2016) was used as the reference mitochondrial genome for the Asian *D. pulex*. The mitochondrial reference genome for *D. obtusa* clade I was derived from the *D. obtusa* whole-genome sequencing database (Ye Z, Lynch M, in preparation SAMN12816670). Due to the absence of existing mitochondrial genomes for the remaining two clades, we generated them de novo; to this end, reads from three high-coverage clones from each of *D. obtusa* clade II and European *D. pulex* were pooled, and mitochondrial genomes constructed using MITObim (Hahn et al. 2013).

### Read Mapping and Clone Filtering

To guarantee high-quality base calling, we remove clones with mean nuclear genome coverage over sites <3× or a total coverage of mitochondrial genome <100×. We filtered clones with possible laboratory contamination using goodness-of-fit values from MAPGD (Ackerman et al. 2017), that is, any clone with goodness-of-fit values across the genome <−0.15 was removed from the analyses. Both *D. pulex* and *D. obtusa* could engage in sexual reproduction, at least once per year (Hebert and Finston 1996a,b; Innes 1997). To maximize the likelihood of getting offspring from sexual reproduction so that each individual represents a unique genotype, we removed closely related individuals in *D. pulex* and *D. obtusa* (e.g., pairs of full sibs that might have hatched from a single resting egg) using the relatedness command of MAPGD (Ackerman et al. 2017) and kept only the clone with the highest coverage in any cluster with relatedness estimate >0.125. Because *D. pulicaria* normally inhabit in permanent lakes and do not engage in sexual reproduction, we did not apply relatedness filtering for *D. pulicaria*.

To avoid potential mapping bias caused by nuclear insertions of mitochondrial DNA sequences (Numts) (Li et al. 2012), we searched for Numts by applying BLAST to the nuclear genomes of *D. pulex*, *D. pulicaria*, and *D. obtusa* using mitochondrial genomes from each of the corresponding clades. Regions with *e*-values <10^−5^ were defined as Numts. Whole-genome sequencing reads for each clone were trimmed using Trimmomatic (Bolger et al. 2014) with default settings and mapped to the clade-specific mitochondrial reference genome following the pipeline from MToolBox 1.1 (Calabrese et al. 2014). Reads that mapped to regions in the mitochondrial genome that were orthologous to the previously identified Numts were then removed from subsequent analyses using Samtools (Li and Durbin 2009).

### Inferring Heteroplasmy

Variant call files were generated using MToolBox (v1.1) (Calabrese et al. 2014) requiring Phred quality scores ≥30. We focused on heteroplasmic sites with single-base substitutions, filtered candidates for heteroplasmy using the following criteria: (i) to minimize potential contributions from sequencing errors, only sites with lower limit of the confidence interval of the heteroplasmy fraction ≥0.01 were used; (ii) candidate sites with >2 alternate alleles (a very rare situation) were removed; (iii) heteroplasmic sites supported by <5 reads were removed; (iv) candidate heteroplasmic sites were removed if they contained a potential indel within 3 bp of the flanking sequence in each direction; and (v) heteroplasmic sites were required to have a resequencing error profile of the data (DREEP) quality score ≥10 (Li and Stoneking 2012).

### Testing for the Complexity of Heteroplasmy and Phasing Haplotypes

To determine whether heteroplasmic individuals contain more than two haplotypes, we evaluated whether the read coverage for the relevant minor alleles was consistent with a binomial sampling distribution, as expected if a clone has only two mitochondrial haplotypes. We further checked for unimodality using *χ*^2^ goodness-of-fit test by calculating: (i) the mean minor-allele frequency (MAF) for each heteroplasmic clone; (ii) from this information, the expected read counts for each minor allele; and (iii) *χ*^2^ values based on observed and expected read counts.

To obtain evidence of recombination between haplotypes, we performed multiple tests. Linkage-disequilibrium measures, *r*^2^, were estimated using the formula described in Hill and Robertson (1968). The fraction of pairs of informative sites that passed the four-gamete tests (FGTs, Hudson and Kaplan 1985), *F*(*D*′) was calculated using the number of pairs of informative sites that passed the FGTs divided by the total pairs of sites. To avoid signals from sequencing error, we only used minor alleles that appeared in ≥3 haplotypes. Recombination tests based on the pairwise homoplasy index (PHI, Bruen, et al. 2006), Max *χ*^2^ (Maynard Smith and Smith 1992), and neighbor similarity score (NSS, Jakobsen and Easteal 1996) were also performed using the PhiPack package (Bruen 2005) with default parameters. Because D-loop regions (sites 14645–15333) in *Daphnia* are known to contain mutational hotspots (Xu et al. 2012), and also present alignment difficulties, which might lead to false predictions of recombination (Innan and Nordborg 2002), we removed sites from such regions from our analysis.

We applied the *pairwise* program with default parameters in LDhat (McVean et al. 2002) to estimate the minimum number of recombination events in population RAP, given that it is the only population exhibiting compelling evidence of recombination. To estimate the mitochondrial genome recombination rate, we first modified the LDhat lookup table to use a population mutation rate of 0.01, which is roughly compatible with the data reported on silent-site diversity below, and the haplotype number in the corresponding population; then, the *interval* program from LDhat was used to infer the recombination rate using parameters known to be suitable for *D. pulex* data (i.e., block penalty = 5; number of iterations = 1,000,000; and number of updates between samples = 3,500; Urban 2018). LDhot (Auton et al. 2014) was used to detect hotspot regions in the mitochondrial genome, using the output derived from LDhat as input and simulating 1,000 random data sets as null expectations.

### Construction of Phylogenetic Trees

To construct phylogenetic trees with information from heteroplasmic sites, we first phased clones that are predicted to have two haplotypes (i.e., all major alleles go to one haplotype and all minor alleles go to the other). From the results of the *χ*^2^ goodness-of-fit test (supplementary file, Supplementary material online), only clones with >0.95 probability of support for the null hypothesis of only two haplotypes in a clone, were phased into two haplotypes. For the remaining clones, the consensus sequence for each clone is constructed using Samtools (Li 2011) with the following command: (i) *samtools mpileup -q 30 -Q 20 –uf reference_genome.fa clone.bam | bcftools call –mv –V indels | bcftools filter –s LowQual –i ‘%QUAL>20 & DP>=100’ -Oz -o clone_vcf.gz* and (ii) *cat reference_genome.fa | bcftools consensus clone_vcf.gz > clone_cns.fa*. *Daphnia magna* is used as an outgroup in the phylogenetic tree and the sequence is downloaded from National Center for Biotechnology Information (NCBI) (accession number: NC_026914.1). MEGA-X (Kumar et al. 2018) was used to align the sequences and construct a neighbor-joining (NJ) tree with 1,000 bootstrap replicates (distance matrix provided in supplementary file, Supplementary Material online). To construct the maximum-likelihood (ML) tree, we first selected the best substitution model using ModelFinder (Kalyaanamoorthy et al. 2017). Then, the ML tree was constructed with IQ-TREE2 (Minh et al. 2020) with 1000 Ultrafast Bootstrap (Minh et al. 2013).

### mtDNA Population-Genetic Analysis

Mitochondrial and nuclear genomes have different genetic codes, and throughout we used the invertebrate mitochondrial genetic code for *Daphnia*. Nucleotide sequences for the 13 protein-coding genes were extracted from the consensus sequences based on the gene annotation file NC_000844.1, aligned for each gene using the MAFFT (multiple alignment program for amino acid or nucleotide) program with default parameters (Katoh and Standley 2013). We estimated within-population diversity at nonsynonymous and synonymous sites, *π*_n_ and *π*_s_, for each of the 13 mitochondrial protein-coding genes. For a particular biallelic site in a gene, *π* was estimated as 2*pq*, where *p* and *q* are the major and minor allele frequencies. For rare triallelic sites, *π* was estimated as 2(*pq + pr + qr*), where *p*, *q*, and *r* are the frequencies for the three alleles. Mean within-population *π*_n_ and *π*_s_ over the 13 genes were used to estimate mean *π*_n_/*π*_s_, to avoid extreme ratio values resulting from sampling variance, with the variance of mean *π*_n_/*π*_s_ being obtained from the Delta-method equation for the variance of a ratio (A1.19b, in Lynch and Walsh 1998).

In addition, we calculated between-population diversity, Φ, using the framework of Weir and Cockerham (1984), as in Maruki et al. (2022). For each site, Φ was calculated as *H*_t_ − *H*_s_, where *H*_t_ is the total metapopulation diversity obtained using the average allele frequencies over all populations, and *H*_s_ is the mean of the population diversity estimates weighted by the clone number from each population. Final estimates of Φ were then calculated by averaging over all sites within each gene, and standard errors for Φ_n_/Φ_s_ were estimated with the Delta-method formulation (A1.19b, in Lynch and Walsh 1998).

To measure population subdivision within clades, we calculated the fixation index *F*_ST_ (Wright’s 1951) using the framework of Weir and Cockerham (1984) for each biallelic site in the mitochondrial and nuclear genomes, as described in Maruki et al. (2022). Because *F*_ST_ estimates are biased when MAFs are <0.1 (Maruki et al. 2022), we restricted our analysis on sites with MAF > 0.1. Then, the overall estimates of *F*_ST_ were obtained by taking averages over all biallelic sites (Berg and Hamrick 1997) in the mitochondrial and nuclear genomes, respectively.

To quantify divergence between phylogenetic clades, we estimated synonymous and nonsynonymous substitutions per nonsynonymous and synonymous sites, *d*_n_ and *d*_s_, for each of the 13 mitochondrial protein-coding genes. To be consistent with parallel nuclear-genome analyses (Maruki et al. 2022), as an outgroup we used the same *D. obtusa* clone from haplogroup I in Maruki et al. (2022) to calculate between species divergence. Synonymous and nonsynonymous substitutions and the numbers of potentially synonymous and nonsynonymous sites were estimated following the modified Nei–Gojobori method (Zhang et al. 1998), where the transition and transversion ratio of 7.3:1 for the mtDNA of NA *D. pulex* was obtained from Tucker (2009). Mean *d*_n_ and *d*_s_ for each functional category (e.g., electron-transport chain complexes I, III, and IV) were obtained by averaging the values from all genes in the category, and mean *d*_n_/*d*_s_ was obtained by dividing mean *d*_n_ by mean *d*_s_, and again, the variance of *d*_n_/*d*_s_ was obtained with the Delta-method equation for the variance of a ratio (A1.19b, in Lynch and Walsh 1998).

To infer genes potentially under positive selection, we evaluated the neutrality index (NI) for 10 Midwest *D. pulex* populations, based on ratios of within-species diversities and among-species divergence (Betancourt et al. 2012). Specifically, we used NI = (Π_n_/Π_s_)/(*d*_n_/*d*_s_), where Π_x_ =*π*_x_ + Φ_x_ is the total metapopulation diversity (with x = s or n), with the variance of NI again obtained with the Delta-method equation. With this estimator, NI < 1.0 implies positive selection at the divergence level, whereas NI > 1.0 suggests purifying selection.

## Results

### Mitochondrial Heteroplasmy is Widespread in *D. pulex*, *D. pulicaria*, and *D. obtusa*

This study relies on whole-genome sequencing data from 1,804 *Daphnia* clones: 1,359 NA *D. pulex*; 30 European *D. pulex*; 42 Asian *D. pulex*; 201 NA *D. pulicaria*; and 172 NA *D. obtusa* (table 1). The mean depth of sequence coverage per clone for the mitochondrial genome was ∼387× per individual, ranging from 100× to 6,860× (supplementary fig. S1, Supplementary Material online). Multiple observations reveal a high prevalence of mitochondrial heteroplasmy in the three *Daphnia* species: 99.4% of the *D. obtusa* and 96.5% of the *D. pulicaria* clones contain heteroplasmic sites, and 65.5% of the sexual and 91.7% of the obligately asexual *D. pulex* from NA contain such sites (table 1). In total, 1,800 heteroplasmic sites were found in *D. obtusa* clones, 1,429 in NA sexual *D. pulex* (556 sites from obligately asexual *D. pulex*), and 251 in *D. pulicaria* clones. These heteroplasmic sites appear to be evenly distributed across the entire mitochondrial genome (supplementary table S2 and supplementary fig. S2, Supplementary Material online).

**Table 1.** Summary of Heteroplasmic Mutations for 1804 Clones from *D. pulex*, *D. pulicaria*, and *D. obtusa*.

Population ID	Sample size	Heteroplasmic clone	Heteroplasmic sites	Minor allele frequencies
US *D. pulex*				
BUS	88	59 (0.67)	2.75 [0.25]	0.116 [0.010]
CHQ	93	56 (0.60)	1.95 [0.29]	0.076 [0.011]
EB	77	29 (0.38)	8.86 [2.33]	0.068 [0.016]
KAP	79	65 (0.82)	2.80 [0.17]	0.067 [0.005]
LPA	87	59 (0.68)	4.29 [1.38]	0.078 [0.009]
LPB	84	56 (0.67)	7.54 [2.73]	0.085 [0.008]
NFL	89	63 (0.71)	2.91 [0.72]	0.094 [0.012]
PA	440	280 (0.64)	2.78 [0.26]	0.073 [0.004]
POV	64	47 (0.73)	6.70 [1.77]	0.073 [0.010]
TEX	66	50 (0.76)	12.18 [2.91]	0.072 [0.009]
Asex	36	33 (0.92)	21.55 [10.86]	0.053 [0.007]
OA (Oregon)	66	48 (0.73)	10.56 [5.09]	0.112 [0.012]
SH (Oregon)	90	66 (0.73)	2.30 [0.19]	0.087 [0.010]
Non-US *D. pulex*				
BEL (Europe)	30	29 (0.97)	1.38 [0.11]	0.073 [0.009]
SZH (Asia)	42	42 (1.00)	22.23 [0.75]	0.177 [0.004]
*D. pulicaria*				
BRA	79	73 (0.92)	2.18 [0.13]	0.033 [0.002]
CLO	60	60 (1.00)	13.37 [1.38]	0.046 [0.003]
TF	62	61 (0.98)	9.77 [0.90]	0.065 [0.010]
*D. obtusa*				
EBG	67	66 (0.99)	27.03 [2.31]	0.054 [0.004]
PYR	43	43 (1.00)	28.74 [3.96]	0.055 [0.005]
RAP	62	62 (1.00)	302.00 [34.40]	0.049 [0.003]

Numbers within parentheses and brackets are fractions and standard errors. Mean minor-allele frequencies are calculated within each heteroplasmic clone and then averaged across all such clones.

**Fig. 1.**
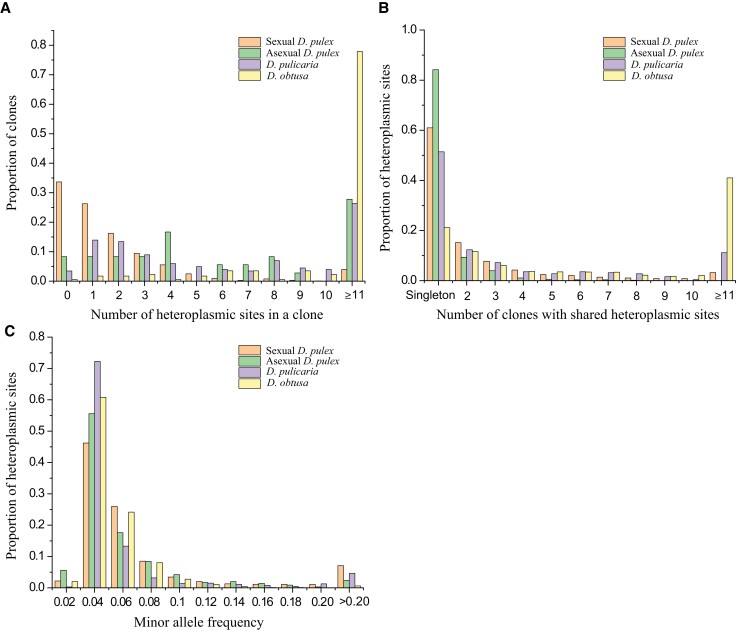
Distributions of heteroplasmic variants within *Daphnia* clones. (*A*) Proportions of clones carrying specific numbers of heteroplasmic sites, including the homoplasmic classes. (*B*) Heteroplasmic sites are shared.0 across clones; this is equivalent to the site-frequency spectrum, with singletons denoting heteroplasmic sites found in just one clone. (*C*) Histograms for mean minor-allele frequencies of heteroplasmic sites within all heteroplasmic clones.

The number of heteroplasmic sites per heteroplasmic clone in *D. obtusa*, mean = 127.2 and median = 29.0, is significantly higher than that in heteroplasmic *D. pulicaria* clones, mean = 8.0, median = 6.0 (*P* < 0.0001, Mann–Whitney *U* test). Moreover, obligately asexual *D. pulex* (mean = 21.5, median = 6.0) have significantly more heteroplasmic sites than sexual *D. pulex* from NA, mean = 4.4, median = 2.0 (*P* < 0.0001, Mann–Whitney *U* test); 80.2% of clones in *D. obtusa* and 30.3% in *D. pulicaria* have ≥10 heteroplasmic sites, while the proportion for NA *D. pulex* is only 4.1% (27.8% for asexual clones) (fig. 1*A*). We found that 78.8% of the heteroplasmic sites in *D. obtusa* are shared by ≥2 isolates, whereas 39.0% (15.8% for asexual isolates) and 48.6% of the heteroplasmic sites in sexual *D. pulex* and *D. pulicaria* are shared across isolates (fig. 1*B*). We further examined sites that were heteroplasmic in parallel across different populations within each species, revealing that such configurations were present for 15.4% of heteroplasmic sites in *D. obtusa*, 23.6% in sexual *D. pulex* (15.5% for asexual isolates), and 24.3% in *D. pulicaria*. Based on nuclear-genome analyses for *D. pulex* (Maruki et al. 2022), all of the sampled populations consist of individuals with distinct nuclear genomes (i.e., are not clonemates), so the presence of shared heteroplasmy across populations suggests that heteroplasmy is common in *Daphnia* species, as inferred from detection in multiple populations. We further examined heteroplasmic sites that are minor alleles in one clone but appear as major alleles in other clones. We found that each population has 1–80 such incidents except for population BEL (supplementary table S3, Supplementary Material online).

To further ascertain the basis of mitochondrial heteroplasmy at the individual level (e.g., the number of potential haplotypes carried within individuals), we examined the MAFs of heteroplasmic sites within individuals. At very low heteroplasmy levels, it becomes impossible to distinguish between sequencing errors and the true mtDNA sequence polymorphism due to the baseline error rates of high-throughput sequencing, and previous studies using high-depth sequencing have generally used a heteroplasmy threshold from 1% to 2% (Goto et al. 2011; Guo et al. 2012; Ng et al. 2020). Our results indicate that very-low-frequency (MAF < 0.02) heteroplasmic sites are very rare (asexual *D. pulex*: 5.6%; sexual *D. pulex*: 2.2%; *D. pulicaria*: 0.3%; *D. obtusa*: 2.0%) in all *Daphnia* species, although most are <0.20 (fig. 1*C*). The proportions of heteroplasmic sites with MAF > 0.20 are 7.1% in sexual *D. pulex* (2.4% for asexual *D. pulex*), 4.6% in *D. pulicaria*, and 0.6% in *D. obtusa*, respectively (fig. 1*C*). The mean MAF for sexual and asexual *D. pulex*, *D. pulicaria*, and *D obtusa* are 0.067, 0.051, 0.051, and 0.042.

### Evidence for Recombination

Although it is generally thought that animal mitochondria are essentially nonrecombining (Berlin and Ellegren 2001; Berlin et al. 2004; Lynch 2007), such rampant heteroplasmy implies a significant opportunity for recombination. We started by looking for potential recombination events within clones. For each heteroplasmic clone, we searched for reads covering ≥2 heteroplasmic sites so that the haplotypes could be inferred directly from the reads. We applied FGTs (Hudson and Kaplan 1985) to check for presence of the four possible haplotypes for pairs of biallelic sites. In the absence of recombination, such configurations require at least three independent mutational events, two of which must be parallel. As the probability of the latter event will typically be <10^−3^ given the amount of silent-site variation in these populations (table 2), a recombination event can be reasonably inferred if all four haplotypes are present, yet in no case did direct analysis of the short-read data support the occurrence of recombination.

**Table 2.** Measures of Genetic Diversity for Populations from *D. pulex*, *D. pulicaria*, and *D. obtusa*.

Population ID	*π*_n_	*π*_s_	*π*_n_/*π*_s_	Effective population size (*N*_e_)
NA *D. pulex*				
BUS	0.0000 (0.0000)	0.0002 (0.0001)	0.1191 (0.0156)	730
CHQ	0.0001 (0.0000)	0.0010 (0.0002)	0.1256 (0.0106)	3,650
EB	0.0009 (0.0005)	0.0076 (0.0010)	0.1227 (0.0187)	27,737
KAP	0.0001 (0.0000)	0.0014 (0.0003)	0.0704 (0.0084)	5,109
LPA	0.0021 (0.0003)	0.0187 (0.0019)	0.1132 (0.0058)	68,248
LPB	0.0025 (0.0005)	0.0230 (0.0032)	0.1066 (0.0076)	83,942
NFL	0.0010 (0.0002)	0.0107 (0.0015)	0.0897 (0.0065)	39,051
PA	0.0009 (0.0003)	0.0046 (0.0010)	0.1966 (0.0233)	16,788
POV	0.0015 (0.0004)	0.0134 (0.0022)	0.1146 (0.0105)	48,905
TEX	0.0009 (0.0002)	0.0102 (0.0021)	0.0921 (0.0070)	37,226
OA (Oregon)	0.0009 (0.0005)	0.0047 (0.0011)	0.1848 (0.0304)	17,153
SH (Oregon)	0.0019 (0.0004)	0.0108 (0.0021)	0.1743 (0.0133)	39,416
Non-NA *D. pulex*				
BEL (Europe)	0.0007 (0.0002)	0.0089 (0.0014)	0.0784 (0.0064)	32,482
SZH (Asia)	0.0019 (0.0007)	0.0149 (0.0019)	0.1293 (0.0139)	54,380
NA *D. pulicaria*				
BRA	0.0000 (0.0000)	0.0000 (0.0000)	0.1293 (0.0401)	0
CLO	0.0031 (0.0006)	0.0355 (0.0023)	0.0864 (0.0049)	129,562
TF	0.0001 (0.0001)	0.0021 (0.0004)	0.0526 (0.0083)	7,664
NA *D. obtusa*				
EBG	0.0010 (0.0008)	0.0020 (0.0011)	0.4987 (0.1326)	7,299
PYR	0.0012 (0.0003)	0.0203 (0.0023)	0.0592 (0.0045)	74,088
RAP	0.0018 (0.0006)	0.0199 (0.0022)	0.0902 (0.0089)	72,628

Within-population nucleotide diversity is the average number of nucleotide differences per site between randomly chosen sequences. The nonsynonymous and synonymous variation within populations is denoted by *π*_n_ and *π*_s_, respectively. Numbers in parentheses are standard errors. The effective population size (*N*_e_) for *D. pulex* populations was estimated as *π*_s_/2*μ* (as described in the Materials and Methods). The *N*_e_ for *D. pulicaria* and *D. obtusa* are estimated using the mutation rate for *D. pulex*. NA, North America.

Because the preceding analysis can only detect recombination events within single reads of length 100–150 bp, it remains possible that recombination occurs between more distant sites. To further infer the presence of multiple haplotypes within each heteroplasmic clone, we performed a *χ*^2^ goodness-of-fit test for the MAFs. If there are only two mitochondrial haplotypes within a heteroplasmic clone, the MAFs for the heteroplasmic sites should follow binomial distribution. In an examination of 447 clones containing ≥5 heteroplasmic sites, 64 were predicted to have more than two haplotypes (supplementary file, Supplementary Material online), thereby suggesting recombination within heteroplasmic clones. However, due to the limited lengths of the short reads, we were unable to recover the precise haplotypes.

We next sought evidence for recombination among haplotypes within each population. For heteroplasmic clones with just two mitochondrial haplotypes, we constructed the two haplotypes based on allele frequency, that is, assigning alleles with higher frequencies to the major haplotype and those with lower frequencies to the minor haplotype. For all remaining heteroplasmic clones (inferred to have >2 haplotypes), only haplotypes from major alleles were used. Multiple tests were then performed to search for signals of recombination using the entire pool of haplotypes from each population.

**Fig. 2.**
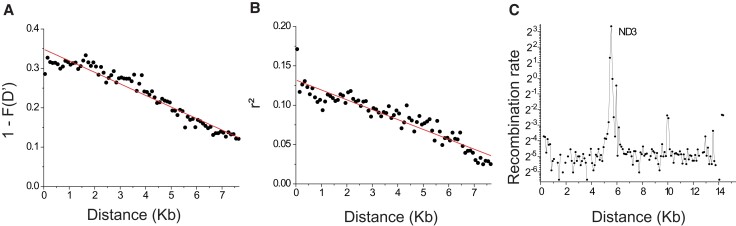
Linkage-disequilibrium profile for population RAP (from *D. obtusa*). (*A*) Relationship between the fraction of pairs of informative markers for which the four possible haplotypes are present, *F*(*D*′), and the physical distance between biallelic sites (slope = (−2.933 ± 0.009) × 10^−5^, *P* < 0.0001). (*B*) Correlation between linkage-disequilibrium measures, *r*^2^, and physical distance between sites (slope = (−1.255 ± 0.053) × 10^−5^, *P* < 0.0001). The data were binned into 100-bp windows according to distance, and the average *r*^2^ for each bin is plotted. Degree of freedom is *n* − 1, where *n* is the total number of bins. For example, for all pairs of biallelic sites with distance between 1 and 100 bp, an average *r*^2^ is calculated and plotted, and so on for 101–200 bp, 201–300 bp, etc. For each pair of biallelic sites, minor alleles were required to appear >2 times in the pooled haplotypes to be used in the analysis. (*C*) Population-level recombination rate, 2*N*_e_*c*, estimated from LDhat (McVean et al. 2002) The *x*-axis denotes the location on the mitochondrial genome, and the *y*-axis is the population recombination rate (per kb per generation).

First, the application of FGTs yielded evidence for recombination in 15 of the 20 populations (supplementary table S4, Supplementary Material online), although most populations had just a small fraction (<10%) of pairs of sites passing FGTs, except for populations EBG (23%) and RAP (25%). Unlike the situation with linear chromosomes, two breaks must occur between flanking markers to induce a recombination event in circular mitochondrial genomes. The size distribution of exchange segments is unknown, but theory indicates that the magnitude of linkage disequilibrium (LD) should increase nearly linearly up to the distance equivalent to the mean exchange-segment length, and thereafter level off (Wiuf 2001). Therefore, we evaluated the relationship between the fraction of pairs of informative markers for which the four possible haplotypes are present, *F*(*D*′), and the physical distance between sites. Applying this approach to each population, just one (RAP, from *D. obtusa*) showed a negative correlation of 1 – *F*(*D*′) with distance (fig. 2*A*; supplementary fig. S3, Supplementary Material online), and even after pooling haplotypes from all populations within each species, only in the case of *D. obtusa* did this approach provide evidence of recombination (supplementary fig. S3, Supplementary Material online), and the latter pattern was eliminated after removing the RAP haplotypes. Notably, the two haplotypes contained within RAP clones are deeply divergent (1.970 ± 0.224%), suggesting an origin by paternal leakage rather than accumulation of new mutations as it takes thousands of generations of coexistence for new mutations to results in 2% divergence between the major and minor haplotypes, which is unlikely the case in *Daphnia* due to rapid within-individual sorting.

Second, we evaluated the relationship between LD, calculated by *r*^2^ (Hill and Robertson 1968), and the distance between sites for each population. Again, no evidence of recombination emerged, except for a linear association in population RAP (fig. 2*B*; supplementary fig. S4, Supplementary Material online), consistent with the results noted above. A negative correlation between *r*^2^ and distance was also observed in pooled *D. obtusa* (supplementary fig. S4, Supplementary Material online), although this is again caused by the inclusion of RAP (supplementary fig. S4, Supplementary Material online). We searched for further signals of recombination within population RAP with three additional tests: Max *χ*^2^ (Smith 1992), NSS (Jakobsen and Easteal 1996), and PHI(Bruen et al. 2006). Both the Max *χ*^2^ and NSS tests have been found to be reliable for detecting recombination in mtDNA (Posada 2002), and both generated a recombination signal in the RAP population (probability of no recombination <0.0001 in both cases). Results from the PHI test, which is thought to control for both mutational hot spots and population growth (Bruen et al. 2006), also implied recombination in RAP (*P* < 0.0001). Thus, although not entirely independent, every recombination test applied infers mitochondrial recombination within the RAP *D. obtusa* population.

Finally, based on the formula in Hudson and Kaplan (1985), the minimum number of recombination events in RAP is predicted to be 34, and from LDhat (McVean et al. 2002) results, the average population recombination rate (2*N*_e_*c*) is estimated to be 0.068/kb/generation in RAP. This average level of recombination is >100× lower than the inferred rate in the nuclear genome of *D. pulex* (Lynch et al. 2017). However, a potential recombinational hotspot was detected within the mitochondrial gene for nicotinamide adenine dinucleotide hydrogen dehydrogenase 3 (fig. 2*C*), suggesting that the high signal of mitochondrial recombination may be a consequence of an unusual feature in this one population.

### Phylogeny and Gene Flow Within and Across *Daphnia* Species

**Fig. 3.**
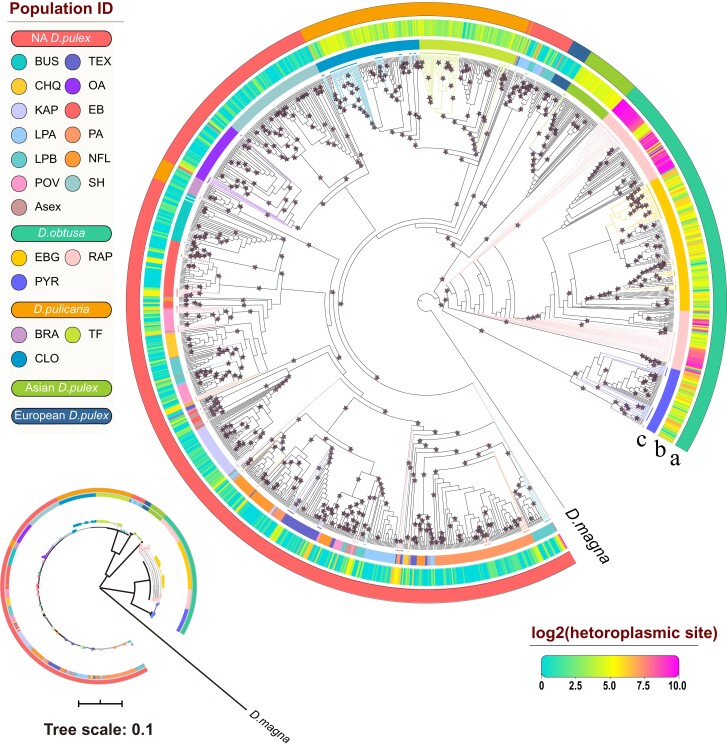
Mitochondrial phylogeny of the *D. pulex*, *D. pulicaria*, and *D. obtusa* clones based on maximum-likelihood analysis of the full-length mitochondrial sequences. *Daphnia magna* was used as an outgroup. Clones with two haplotypes are phased using allele frequencies, that is, assigning all major alleles to one haplotype, and all minor alleles to the other. Haplotypes constructed from minor alleles are marked by colored branches and solid circles at the tip of the corresponding branches. Arc (*a*) shows the color-coded species, with species name listed on the left panel; Arc (*b*) indicates the density of the heteroplasmic sites within each clone; Arc (*c*) shows the color-coded populations within each species. Stars indicate bootstrap values >75%. Inset on the bottom left shows the branch length of the major clades.

To infer the existence of gene flow within and between *Daphnia* species, we constructed ML and NJ trees (fig. 3; supplementary fig. S5, Supplementary Material online) for *D. pulex*, *D. pulicaria*, and *D. obtusa* using full-length mitochondrial sequences. As noted above, clones with two haplotypes were phased using allele frequencies, and in all three *Daphnia* species, minor haplotypes always cluster with major haplotypes from the same population (fig. 3; supplementary fig. S5, Supplementary Material online). In both ML and NJ trees, *D. obtusa* falls outside of all *D. pulex* and *D. pulicaria*, consistent with previous analyses (Hebert and Finston 1996a; Cornetti et al. 2019). For *D. obtusa*, both types of trees revealed that there are at least two genetically distinct haplogroups (fig. 3; supplementary fig. S5, Supplementary Material online). Haplogroup I consists of clones from the RAP and EBG populations, whereas haplogroup II contains only clones from the PYR population. Our analysis, based on the entire mitochondrial genome, supports the hypothesis that NA *D. obtusa* consists of two deeply divided clades (sequence divergence: 13.4%), consistent with previous findings (supplementary fig. S6, Supplementary Material online; Penton et al. 2004). Asian *D. pulex* is placed as an independent clade compared with other *D. pulex*, and European *D. pulex* is a sister lineage to all NA *D. pulicaria* and *D. pulex*, as inferred by Crease et al. (2012) (fig. 3; supplementary fig. S5, Supplementary Material online). On the ML tree, all clones from *D. pulicaria* population BRA cluster with NA Midwest *D. pulex* populations (fig. 3), while on the NJ tree all BRA clones and a small number of clones from CLO also cluster with NA Midwest *D. pulex* populations (supplementary fig. S5, Supplementary Material online), suggesting introgression between at least *D. pulicaria* (BRA) and Midwest *D. pulex*, and/or long-lived ancestral polymorphism. The two Oregon *D. pulex* populations (OA and SH) form a separate clade (fig. 3), consistent with the conclusion of Crease et al. (1997, 2012) that a US West Coast *D. pulex* clade diverged from more eastern *D. pulex* as a consequence of geographic isolation by glaciation (Crease et al. 1997). Within the 10 NA Midwest *D. pulex* populations, shared haplotypes (haplotypes from one population cluster more closely with haplotypes from other populations) are common (fig. 3; supplementary fig. S5, Supplementary Material online).

To further evaluate the level of population subdivision and gene flow in the 10 Midwest *D. pulex* populations, we estimated pairwise *F*_ST_, a coefficient of gene differentiation. Using all polymorphic sites in the complete mitochondrial genomes, the average *F*_ST_ among this subset of populations is 0.158 ± 0.015 (see pairwise *F*_ST_ in the supplementary file, Supplementary Material online), which is lower than that from the nuclear genomes in these populations (0.248 ± 0.009, Maruki et al. 2022). The level of gene flow, *N*_e_*m* (the effective population number and rate of migration among populations), was calculated following Wright (1965). For a haploid genome such as the mitochondrion, *F*_ST_ = 1/(1 + 2*N*_e_*m*), while in the diploid genome *F*_ST_ = 1/(1 + 4*N*_e_*m*). We estimated that the mean number of migrants per generation, *N*_e_*m*, for the mitochondrion and nuclear genomes are 2.641 and 0.757 (supplementary table S5, Supplementary Material online), both suggest a relatively high level of gene flow within NA *D. pulex*. For the nuclear genome in these populations, pairwise *F*_ST_ exhibits a weak but positive correlation with the geographic distance (Maruki et al. 2022). For the mitochondrial genome, *F*_ST_ is only marginally positively correlated with distance (supplementary fig. S7, Supplementary Material online; Pearson *r* = 0.26; *P* = 0.08). In addition, *F*_ST_ for the mitochondrial genome were found to be significantly associated with *F*_ST_ for the nuclear genome (supplementary fig. S7, Supplementary Material online; Pearson *r* = 0.64; *P* < 0.0001).

To determine the potential gene flow between NA *D. pulex* and *D. pulicaria*, we calculated *F*_ST_ and *N*_e_*m* for both the mitochondrial and nuclear genomes (supplementary table S5 and supplementary file, Supplementary Material online). The *F*_ST_ values averaged across all pairs of populations between NA *D. pulex* and *D. pulicaria* are 0.438 and 0.204, and *Nm* values are 0.321 and 1.945 for the mitochondrial and nuclear genome (supplementary table S5, Supplementary Material online and the supplementary file, Supplementary Material online). Our results revealed a relatively high level of gene flow between NA *D. pulex* and *D. pulicaria*, which is about 42% of that within *D. pulex* for the nuclear genome.

### Magnitude of Purifying Selection in Mitochondrial Protein-Coding Genes

*Daphnia* populations exhibit moderately high levels of within-population sequence variation in the mitochondrion. The diversity at synonymous sites within mitochondrial protein-coding sequences varies from 0.0002 (BUS) to 0.0230 (LPB) (table 2). The correlation between genome-wide mitochondrial and nuclear synonymous-site diversities (obtained from Maruki et al. 2022) among the 10 NA Midwest *D. pulex* populations is not significant (linear regression: *r*^2^ = 0.014, *P* = 0.74). Given the typically uniparental nature of mitochondrial inheritance, this is unsurprising, as the organelle genome effectively represents one degree of freedom relative to the large number of effectively freely recombining and segregating sites in the nuclear genome (essentially all pairs of sites >1 Mb apart; Lynch et al. 2022).

Owing to uniparental, haploid inheritance, the effective population size (*N*_e_) for mitochondrial DNA is expected to be lower than that for nuclear DNA, with a ratio of 1:4 often being assumed under ideal conditions of random mating (Palumbi et al. 2001; Lynch 2007). Letting *N*_e_ = *π*_s_/2*μ*, with *μ* = 1.37 × 10^−7^ site/generation as the *D. pulex* mitochondrial DNA mutation rate (Xu et al. 2012), we obtained estimates of the effective number of mitochondria for each of 10 NA Midwest populations (under the assumption that *π*_s_ reflects drift-mutation equilibrium). Unlike the relatively constant *N*_e_ values observed for the nuclear genomes in these populations, where the average is 640,000 (Maruki et al. 2022), *N*_e_ differed markedly among populations (table 2), ranging from a low of 730 in BUS to a high of 83,942 in LPB, with an average across populations of 32,300. Thus, accounting for diploidy in the nuclear genome, the ratio of the average effective numbers in the mitochondrial versus nuclear genome is just 32,330/1,280,002 = 0.025, far below the expectation of 0.5 (based on the idea that one mtDNA genome and two haploid nuclear genomes contribute to sexual offspring production). Because temporal propagation of these intermittent-pond populations (as well as migration) occurs by resting-egg production, and mating appears to be very close to random within populations (Lynch et al. 2017; Maruki et al. 2022), any demographic bottlenecks are expected to be of similar magnitude for both the mitochondrial and nuclear genomes. Thus, these observations suggest that, relative to the nuclear genome, the mitochondrial genomes of *Daphnia* are subject to much more frequent selective sweeps and/or purging of variation by background selection against deleterious mutations.

For the mitochondrial protein-coding genes in the 10 Midwest *D. pulex* populations, for which we have very substantial mitochondrial- and nuclear-genome data, we calculated a metapopulation-wide NI as (Π_n_/Π_s_)/(*d*_n_/*d*_s_), where Π_x_ =  *π*_x_ + Φ_x_ is the total metapopulation diversity (with x = s or n), and *π* denotes the within-population diversity, and Φ denotes the between-population diversity. We obtained NI = 0.607 (0.199), 1.883 (0.751), and 1.346 (0.628) for the proteins in electron-transport chain complexes I, III, and IV, respectively, and 3.640 (3.223) for ATP synthase (table 3). This suggests a predominance of purifying selection on these mitochondrial proteins.

**Table 3.** Measures of Genetic Diversity for Complexes with Both Mitochondrion-Encoded Genes and Nuclear-Encoded Genes.

Category		Gene number	π_n/_π_s_	*φ*_n/_ *φ*_s_	Π_n_/Π_s_	*d*_n_ */d*_s_	NI	SE(NI)
ETC complex I	nuc encoded	41	0.177	0.108	0.157	0.123	1.272	0.348
	mt encoded	7	0.151	0.133	0.140	0.230	0.607	0.199
ETC complex III	nuc encoded	8	0.136	0.116	0.131	0.106	1.236	0.531
	mt encoded	1	0.168	0.153	0.073	0.087	1.883	0.751
ETC complex IV	nuc encoded	8	0.032	0.199	0.130	0.129	1.004	0.359
	mt encoded	3	0.070	0.077	0.783	0.054	1.346	0.628
ATP synthase	nuc encoded	12	0.092	0.041	0.086	0.124	0.688	0.360
	mt encoded	2	0.680	1.106	0.163	0.215	3.640	3.223

As all of the mitochondrion-encoded protein genes produce products that form complexes with products from nuclear-encoded genes, essential for respiration and ATP production, we estimated NI for the associated nuclear-encoded genes to see if these partners were under unusual forms of selection (table 3). For three of the four complexes, there was a substantial reduction in NI for the nuclear-encoded genes relative to what is seen in the mitochondrion-encoded subunits: NI = 1.272 (0.348), 1.236 (0.531), and 1.004 (0.359) for the proteins in electron-transport chain complexes I, III, and IV, respectively, and 0.688 (0.360) for ATP synthase, with an overall average of 1.050 (0.131), very close to the neutral expectation of 1.0. This average estimate is substantially lower than that observed for the total set of nuclear-encoded genes, 1.521 (0.011), in *D. pulex* (Maruki et al. 2022), but is not a consequence of an elevated rate of the divergence ratio (*d*_n_/*d*_s_) in the former (Maruki et al. 2022). Rather, it is a consequence of a reduced within-species ratio (Π_n_/Π_s_) for the nuclear-encoded, mitochondrion-directed genes (Maruki et al. 2022). The reduction of NI suggests increased selection pressure for the nuclear-encoded respiratory proteins.

## Discussion

### Mitochondrial Heteroplasmy in *Daphnia*

Heteroplasmy, once thought to be rare in the mitochondrion, has been shown recently to be prevalent in humans (Ye et al. 2014; Li et al. 2015; Wei et al. 2019), mice (Burgstaller et al. 2018), and fruit flies (Nunes et al. 2013). Here, using large-scale population-genomic data, we show that population-level mtDNA heteroplasmy is also common in *D. pulex*, *D. pulicaria*, and *D. obtusa*. Approximately 65.5% of isolates of sexual *D. pulex*, 99.4% of *D. obtusa*, and 96.5% of *D. pulicaria* clones exhibit heteroplasmy (table 1). Mitochondrial heteroplasmy has been implicated in many human diseases such as aging, cancer, and late-onset neurodegenerative diseases (Taylor and Turnbull 2005; Wallace 2010; Schon et al. 2012). Heteroplasmy in mouse can cause reduced activity, accentuated stress response; and cognitive impairment (Sharpley et al. 2012). For pathogenic mitochondrial heteroplasmy to cause diseases in human or mouse, the proportion of mutated mtDNA normally needs to exceed a critical phenotypic threshold (in some cases 60–80%) in a fraction of cells (King and Attardi 1989; Boulet et al. 1992). However, not all minor mitochondrial variants need to be strongly deleterious. For example, it has been shown that a de novo heteroplasmic mutation in cows can become fixed within just two or three generations (Olivo et al. 1983). In *Daphnia*, heteroplasmic mutations are often in the frequency range of 0.042–0.067, but in almost every population there are cases in which heteroplasmic mutations are minors in some individuals and majors in others (supplementary table S3, Supplementary Material online), suggesting fast transition between major and minor alleles. Future studies are needed to unravel the genetic consequences of heteroplasmy in *Daphnia*, including the degree to which the rare variants are deleterious.

Heteroplasmy must naturally arise at some low level, as individual mitochondrial genomes generate mutations, with the daughter lineages then segregating out (Stewart and Chinnery 2015). Consistent with endogenous origin from mutation, most *Daphnia* populations have just a few heteroplasmic sites, usually no more than 30 (table 1). However, one *D. obtusa* population (RAP; table 1) consists entirely of heteroplasmic clones carrying substantially divergent mitochondrial haplotypes, differing at an average of 302 sites (table 1). Given the mutation rate for the *Daphnia* mitochondrion, the level of divergence among heteroplasmic haplotypes in this population would likely require tens of thousands of generations of neutral coexistence if due to endogenous origin, which is incompatible with all estimates of heteroplasmy loss in other metazoans being less than a few hundred generations (Lynch 2007). Thus, the extensive mitochondrial heteroplasmy in some *D. obtusa*, and probably in multiple cases in the other species, likely reflects rare biparental-inheritance events involving substantially divergent paternal and maternal lineages. Consistent with this, divergent major and minor haplotypes were found in *D. obtusa* clones, especially in population RAP where the two haplotypes would require tens of thousands of generations of coexistence if due to endogenous origin. Extensive heteroplasmy, likely associated with paternal leakage events, has been implicated in a number of other taxa, including water frogs (*Pelophylax* spp.) (Radojičić et al. 2015), bed bugs (*Cimex lectularius*) (Robison et al. 2015), and in hybrids between the partridges *Alectoris graeca* and *A. chukar* (Gandolfi et al. 2017). Consistent with paternal leakage in RAP, mean divergence between the major and minor haplotypes in RAP clones are ∼10× higher than that in clones from other *D. obtusa* populations (1.970 ± 0.224%; supplementary table S6, Supplementary Material online). Moreover, mean divergence for the minor haplotypes in heteroplasmic RAP clones is much higher than that for the major haplotypes (supplementary table S6, Supplementary Material online), raising the additional possibility that the minor haplotypes have substantially elevated mutation rates or origin from recombination.

### Recombination among *Daphnia* Mitochondrial Genomes

Direct evidence of low levels of recombination among mitochondrial genomes has been suggested for many animal species, for example, nematodes (Lunt and Hyman, 1997), mussels (Ladoukakis and Zouros 2001), fish (Hoarau et al. 2002; Ciborowski et al. 2007), fruit flies (Ma and O’Farrell 2015), and human (Kraytsberg et al. 2004). Using mitochondrial data from public databases, Tsaousis et al. (2005) identified >30 recombination events in the mitochondrial genomes of diverse metazoans, although due to limited sequence information in prior studies, the number of recombination events may have been severely underestimated.

Using high-coverage sequencing data across entire genomes, we find limited evidence of mitochondrial recombination in *Daphnia* species. The one exception is the RAP population of *D. obtusa*, which revealed substantial evidence of recombination via several semi-independent methods of analysis. As the clones in this population have >10× more heteroplasmic sites per clone than any other population, it remains unclear whether the positive results for this population are a consequence of the elevated power of analysis and/or a consequence of unusual recombinogenic activity in a hybrid background.

The average distance between sites with detectable recombination in the RAP population is 4,162 bp (SD = 2,921), which may explain why we did not detect any recombination signal within single reads with lengths in the range of 100–150 bp. In RAP clones, the minor haplotypes are not only divergent from major haplotypes within the same clones (1.97%), but also divergent with existing minor haplotypes from other clones (2.83%; supplementary table S6, Supplementary Material online), raising the issue of their origin. The median-joining haplotype network for the RAP population (supplementary fig. S8, Supplementary Material online) suggests that a few minor haplotypes evolved endogenously via coexisting major haplotypes. However, most of the minor haplotypes in RAP, given their substantial divergence from major haplotypes (supplementary fig. S8, Supplementary Material online), are unlikely solely from mutation or recombination of existing major haplotypes. Instead, their origin must involve recombination with at least one of the existing minor haplotypes. The haplotype network for RAP suggests that within each heteroplasmic clone the two mtDNA haplotypes are just like two alleles (A, a), either of them could recombine with the two alleles in other heteroplasmic clones (B, a). Consistent with this hypothesis, 64 heteroplasmic clones were found to have more than two haplotypes (supplementary file, Supplementary material online), likely caused by the coexistence of two alleles and their recombinants. However, due to the limited lengths of the short reads, we were unable to recover the precise haplotypes and validate if recombination is occurring. The recombinants are supposed to have very low frequencies if generated by recombination between major and minor haplotypes as the MAF is normally ∼5%. To detect the recombinants, we propose to sequence mtDNA to high coverage (e.g., >1,000×) using long reads, so the haplotypes can be directly inferred from reads.

### Divergent Lineages in the *D. pulex* Complex and *D. obtusa*

Our estimated phylogeny supports the hypothesis of allopatric speciation within the *D. pulex* complex (Cristescu et al. 2012; Marková et al. 2013), suggesting that geographic isolation is a major force in shaping the global pattern of mitochondrial diversification. Based on populations across three continents, the mitochondrial phylogenetic tree for the *D. pulex* complex reveals at least three divergent clades (NA *D. pulex/D. pulicaria*, European *D. pulex*, and Asian *D. pulex*; fig. 3). North American *D. pulex* and *D. pulicaria* are sister clades at the nuclear level (Ye et al. 2021), but the mitochondrial haplotypes are interwoven, which could explain discordant phylogenetic trees based on mitochondrial and nuclear genes (Marková et al. 2013; Ye et al. 2021). We find no evidence of recombination between haplotypes in the dominant *D. pulex* and *D. pulicaria* clades in NA. Consistent with Colbourne et al. (1998) and Crease et al. (2012), European *D. pulex* is a sister lineage to all NA *D. pulicaria* and *D. pulex*. *D. pulex* from China, although morphologically indistinguishable from NA *D. pulex*, forms a separate clade, possibly warranting a separate species designation such as *D. mitsukuri* (Ma et al. 2019). Our analysis from three *D. obtusa* populations confirms that NA *D. obtusa* consists of two deeply divided clades, consistent with previous findings (Penton et al. 2004).

### Dramatic Reduction in the Effective Population Size of the Mitochondrion and Magnitude of Purifying Selection in Mitochondrial Protein-Coding Genes

Particularly striking is the substantial difference in effective population sizes of the sites in the mitochondrial versus nuclear genomes in NA *D. pulex*. With the former being ∼2.5% of the latter (and likely also the case in other species; table 2), the efficiency of selection is expected to be much reduced in the mitochondrial genome (for any particular absolute strength of selection per nucleotide site). Nonetheless, the low levels of average *π*_n_/*π*_s,_ Φ_n_/Φ_s_, and *d*_n_/*d*_s_ for the 13 mitochondrial protein-coding genes, averaging 0.25, 0.40, and 0.18 respectively (supplementary file, Supplementary Material online), imply the operation of very strong purifying selection on amino-acid altering mutations in these genes, presumably owing to the substantial deleterious effects of mutations affecting respiratory capacity. Thus, the low effective population sizes associated with the mitochondrial genomes in these populations may largely be a consequence of background-selection effects resulting from the strong purifying selection on amino-acid altering mutations with large effects in the organelle genome.

The selection on mitochondrially encoded genes may be having cascading effects on the nuclear-encoded proteins with which they interact. The NI for the nuclear-encoded respiratory proteins was a fraction 0.56 (0.21) of that for the mitochondrially encoded subunits, suggesting the induction of some positive selection on the former. In principle, such behavior is consistent with the extreme population-genetic environment of the *Daphnia* mitochondrion. With the nearly 40-fold reduction in the effective population size and ∼100-fold increase in the mutation rate relative to the nuclear genome, mutation pressure combined with random genetic drift may facilitate the fixation of mildly deleterious mutations in the mitochondrial genome, imposing selection for compensatory coevolution on the nuclear-encoded genes whose products interact with those from organelle-encoded genes involved in metabolism (the electron-transport chain and ATP synthase subunits). Our results are consistent with prior observations of this sort in the copepod *Tigriopus* (Barreto et al. 2018), vascular plants (Havird et al. 2015), and primates (Osada and Akashi 2012), all of which demonstrate elevated evolutionary rates in nuclear-encoded proteins that interact with mitochondrial-encoded products.

## Supplementary Material

msac059_Supplementary_Data

## Data Availability

The FASTQ files of the raw sequencing data for the 10 Midwest *D. pulex* populations are available at the NCBI Sequence Read Archive (accession number SRP155055), and raw reads for the additional five PA populations are deposited under project ID PRJNA684968. Reads for the Asian *D. pulex*, European *D. pulex*, *D. pulicaria*, and *D. obtusa* can be accessed at NCBI under project ID PRJNA719100. *Daphnia magna* mitochondrial genome is downloaded from NCBI (NC_026914.1). The *D. pulex* genome assembly PA42 v4.1 is available at NCBI GenBank under accession GCA_900092285.2; the *D. pulicaria* genome assembly LK16 under accession number SAMN17106781; and the *D. obtusa* genome assembly under accession number JAACYE000000000. The consensus sequence of the mitochondrial genomes can be accessed at https://osf.io/km8w4/.
