# Supplementary material for: Genetic Diversity, Heteroplasmy, and Recombination in Mitochondrial Genomes of Daphnia pulex, Daphnia pulicaria, and Daphnia obtusa
Source: Mol Biol Evol. 2022 Mar 24;39(4):msac059. doi: 10.1093/molbev/msac059 (PMC9004417; doi:10.1093/molbev/msac059)
Supplement: msac059_Supplementary_Data [file msac059_supplementary_data.zip › Supplementary tables and figures2.docx]

**Table S1.** Sampling locations for all clones used in this study.

| Clones | Species | Latitude (°S) | Longitude (°E) | State, Country |
| --- | --- | --- | --- | --- |
| BUS | *D. pulex* | 40°08′ | -88°13′ | Illinois, USA |
| CHQ | *D. pulex* | 46°40′ | -90°51′ | Wisconsin, USA |
| EB | *D. pulex* | 44°59′ | -93°19′ | Minnesota, USA |
| KAP | *D. pulex* | 40°07′ | -87°44′ | Indiana, USA |
| LPA | *D. pulex* | 42°41′ | -80°25′ | Ontario, Canada |
| LPB | *D. pulex* | 42°41′ | -80°27′ | Ontario, Canada |
| NFL | *D. pulex* | 39°05′ | -84°55′ | Indiana, USA |
| PA | *D. pulex* | 40°13′ | -87°20′ | Indiana, USA |
| POV | *D. pulex* | 42°45′ | -85°21′ | Michigan, USA |
| TEX | *D. pulex* | 42°01′ | -83°4′ | Michigan USA |
| OA | *D. pulex* | 44°25′ | 79°31′ | Oregon, USA |
| SH | *D. pulex* | 44°59′ | 123°01′ | Oregon, USA |
| BEL | *D. ‘pulex’* | 50°03′ | 14°01′ | Czech Republic |
| SZH | *D. ‘pulex’* | 31°16′ | 121°03′ | Suzhou, China |
| BRA | *D. pulicaria* | 45°06′ | -79°31′ | Ontario, USA |
| CLO | *D. pulicaria* | 42°32′ | -85°24′ | Michigan, USA |
| TF | *D. pulicaria* | 46°13′ | -89°31′ | Wisconsin, USA |
| EBG | *D. obtusa* | 40°04′ | -93°41′ | Missouri, USA |
| PYR | *D. obtusa* | 39°12′ | -86°34′ | Indiana, USA |
| RAP | *D. obtusa* | 33°16′ | -88°48′ | Mississippi, USA |

| Regions | Length (bp) | *D. pulex* sex | *D. pulex* asex | *D. pulicaria* | *D. obtusa* |
| --- | --- | --- | --- | --- | --- |
| *Coding* | 10976 | 1205 | 453 | 181 | 1535 |
| ND2 | 990 | 125 (12) | 47 (5) | 20 (2) | 77 (7) |
| COI | 1538 | 133 (1) | 79 (1) | 10 (0) | 321 (2) |
| COII | 679 | 93 (3) | 29 (1) | 3 (0) | 149 (5) |
| ATPase8 | 65 | 2 (0) | 0 (0) | 0 (0) | 17 (2) |
| ATPase6 | 674 | 65 (2) | 19 (1) | 7 (0) | 118 (4) |
| COIII | 789 | 76 (2) | 31 (1) | 7 (0) | 109 (2) |
| ND3 | 353 | 30 (2) | 5 (0) | 4 (0) | 47 (2) |
| ND5 | 1708 | 225 (16) | 54 (4) | 34 (2) | 295 (21) |
| ND4 | 1321 | 159 (12) | 76 (6) | 17 (1) | 196 (15) |
| ND4L | 276 | 34 (2) | 11 (1) | 3 (0) | 31 (1) |
| ND6 | 513 | 66 (8) | 9 (1) | 0 (0) | 24 (3) |
| CytB | 1134 | 100 (2) | 39 (1) | 40 (1) | 117 (3) |
| ND1 | 936 | 97 (6) | 54 (3) | 36 (2) | 34 (2) |
|  |  |  |  |  |  |
| *tRNA* | 1,386 | 115 | 25 | 11 | 144 |
| *rRNA* | 2,067 | 34 | 31 | 33 | 87 |
| *Non-coding*  *coding*  *coding* | 904 | 75 | 47 | 26 | 34 |

**Table S2.** Distribution of the mitochondrial heteroplasmic sites for North American *D. pulex*, *D. pulicaria*, and *D. obtusa*. The numbers in parentheses indicate the number of nonsynonymous

substitutions caused by heteroplasmic sites.

**Table S3**. Number of incidents for minor alleles in one heteroplasmic clone appearing as major alleles in other clones. The total number of incidents for each species is calculated by examining heteroplasmic sites from all clones in a species and it includes both incidents within each population and between clones from different populations.

| Population ID | # Incidents |
| --- | --- |
| NA *D. pulex* | 305 |
| BUS | 4 |
| CHQ | 3 |
| EB | 20 |
| KAP | 1 |
| LPA | 6 |
| LPB | 62 |
| NFL | 11 |
| PA | 38 |
| POV | 52 |
| TEX | 80 |
| OA (Oregon) | 6 |
| SH (Oregon) | 1 |
| SZH (Asia) | 1 |
| *D. pulicaria* | 15 |
| BRA | 3 |
| CLO | 6 |
| TF | 6 |
| *D. obtusa* | 52 |
| EBG | 6 |
| PYR | 1 |
| RAP | 13 |

^\^

**Table S4**. The numbers and percentages (in parentheses) of the pairs of biallelic sites that passed the four-gamete test.

| Population | FGTs (%) |
| --- | --- |
| NA *D. pulex* |  |
| BUS | 1 (5.56) |
| CHQ | 1 (0.06) |
| EB | 47 (0.87) |
| KAP | 3 (6.98) |
| LPA | 314 (1.05) |
| LPB | 484 (1.25) |
| NFL | 135 (0.87) |
| POV | 70 (1.02) |
| PA | 0 (0.00) |
| TEX | 1,398 (9.15) |
| OA (Oregon) | 0 (0.00) |
| SH (Oregon) | 33 (0.44) |
| Non-NA *D. pulex* |  |
| BEL (Europe) | 0 (0.00) |
| SZH (Asia) | 0 (0.00) |
| *D. pulicaria* |  |
| BRA | 0 (0.00) |
| CLO | 551 (0.74) |
| TF | 80 (4.54) |
| *D. obtusa* |  |
| EBG | 1,480 (23.15) |
| PYR | 133 (0.32) |
| RAP | 75,835 (25.01) |

**Table S5**. Fst and Nm (migration rate per generation) for North American (NA) *D. pulex*, and between NA *D. pulex* and *D. pulicaria* populations. Numbers in parentheses are standard errors for estimates from different pairs of populations. F_ST_ estimates were performed using sites with minor allele frequency > 0.1.

| Pairs | mtDNA Fst | mtDNA Nm | Nuclear Fst | Nuclear Nm |
| --- | --- | --- | --- | --- |
| Among 10 NA *D. pulex* populations | 0.158 (0.015) | 2.641 | 0.248 (0.009) | 0.757 |
|  |  |  |  |  |
| *D. pulicaria* vs. *D. pulex* | 0.204 (0.028) | 1.945 | 0.438 (0.007) | 0.321 |
|  |  |  |  |  |

**Table S6**. The mean percentages and standard error (in parentheses) of the divergence within major haplotypes, minor haplotypes, and between major and minor haplotypes from the same clones for *D. obtusa* populations.

| Population | major vs. major | minor vs. minor | major vs. minor |
| --- | --- | --- | --- |
| EBG | 0.163% ± 0.002% | 0.490% ± 0.004% | 0.176% ± 0.022% |
| PYR | 0.570% ± 0.021% | 1.254% ± 0.040% | 0.187% ± 0.026% |
| RAP | 0.577% ± 0.021% | 2.832% ± 0.027% | 1.970% ± 0.224% |

**Figure S1.** Mitochondrial coverage for clones used in this study.


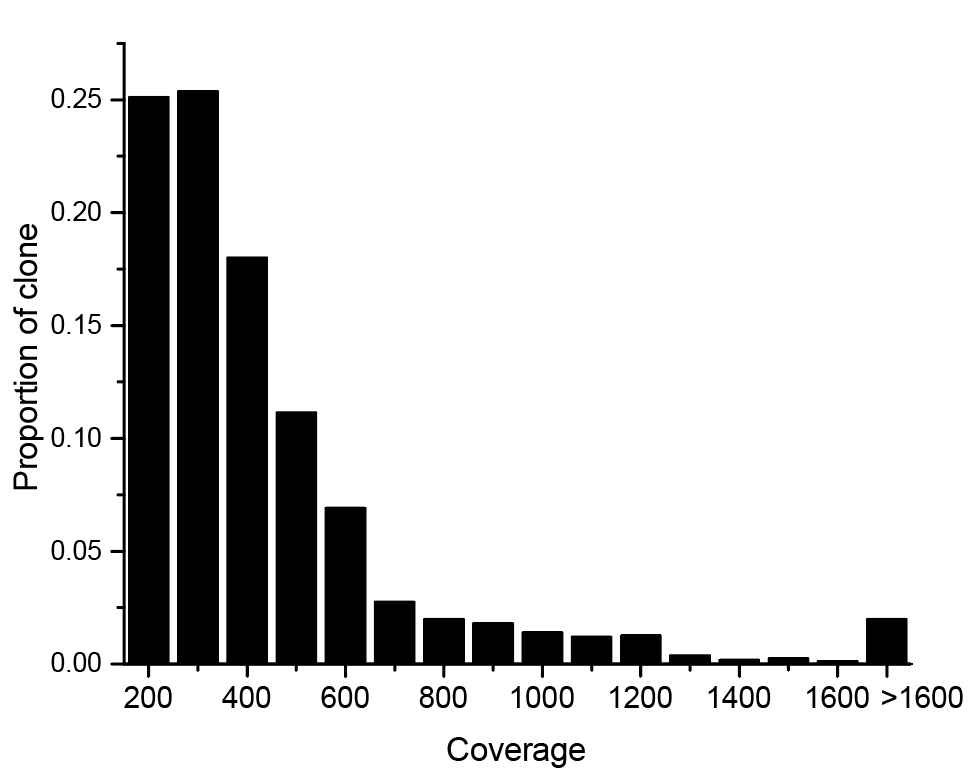


**Figure S2**. Distribution of the heteroplamic sites on the 13 protein-coding genes for the mitochondrial genome of North American *D. pulex*, *D. pulicaria*, and *D. obtusa*. X axis is the length of gene and Y axis denotes the fraction of heteroplamic sites.


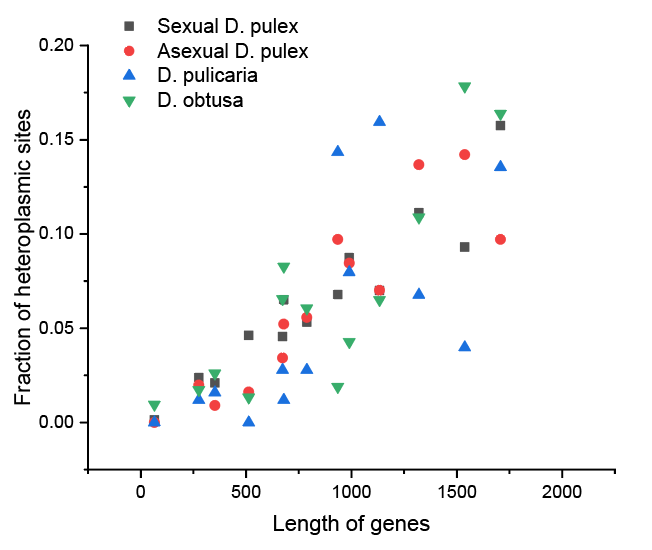


**Figure S3**. Relationship between the fraction of pairs of informative markers for which the four possible gametes are present, F(D′), and the physical distance between sites in each population (Fig. A-K) and in pooled samples for each species (Fig. L-P). Population BUS, CHQ, PA, KAP, OA, BEL, SZH, and BRA have ≤3 pairs of sites passing the FGT and are not plotted. Species level F(D′) is calculated by pooling haplotypes from all populations within each species. The numbers of four-gamete cases are counted for every 100-bp region. For example, if the number of pairs of biallelic sites with distance between 1-100 bp is *n*, and among which *x* pairs passed four-gamete tests, then F(D′) for 1-100 bp is *x/n*.


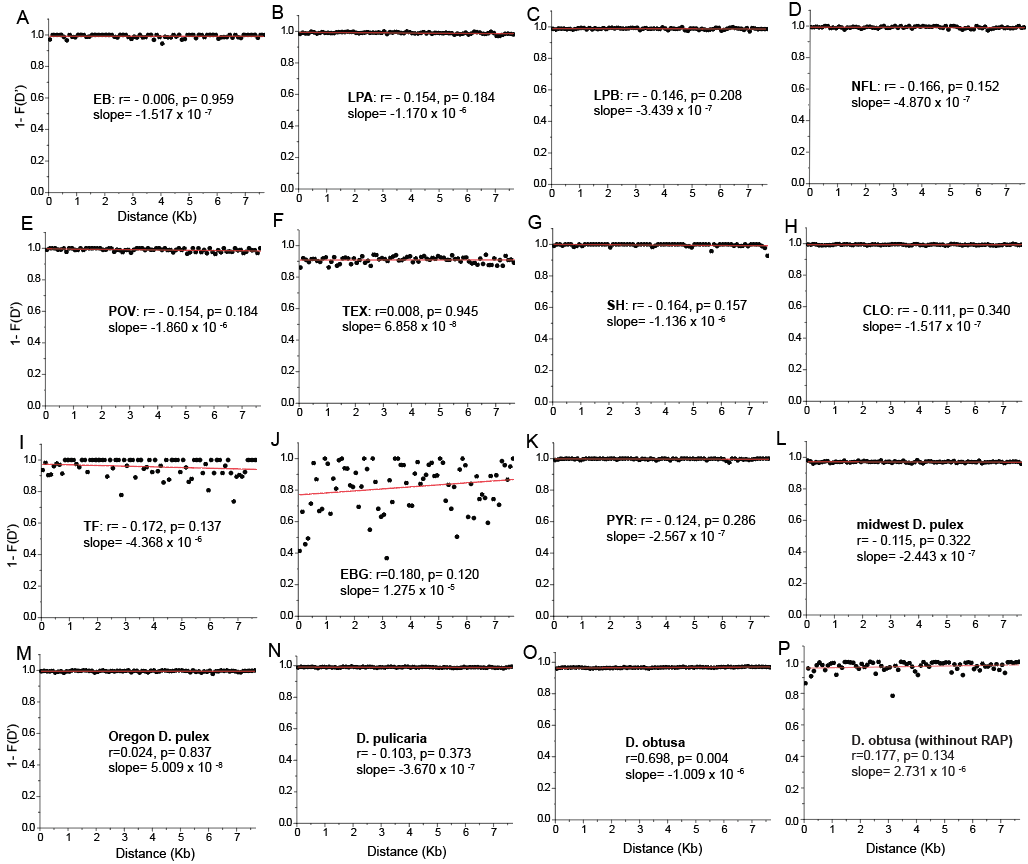


**Figure S4**. Correlation of linkage disequilibrium, r^2^, and physical distance between pairs of biallelic sites in in each population (Fig. A-K) and in each species (Fig. L-P). Species level r^2^ is calculated by pooling haplotypes from all populations within each species. For each biallelic sites, minor alleles were required to appear ≥3 times in a population to be used in the analysis. The data were binned into 100-bp windows according to distance, and the average r^2^ for each bin is plotted. For example, for all pairs of biallelic sites with distance between 1-100 bp, 101-200 bp, 201-300 bp, etc., average r^2^ values were calculated and plotted.


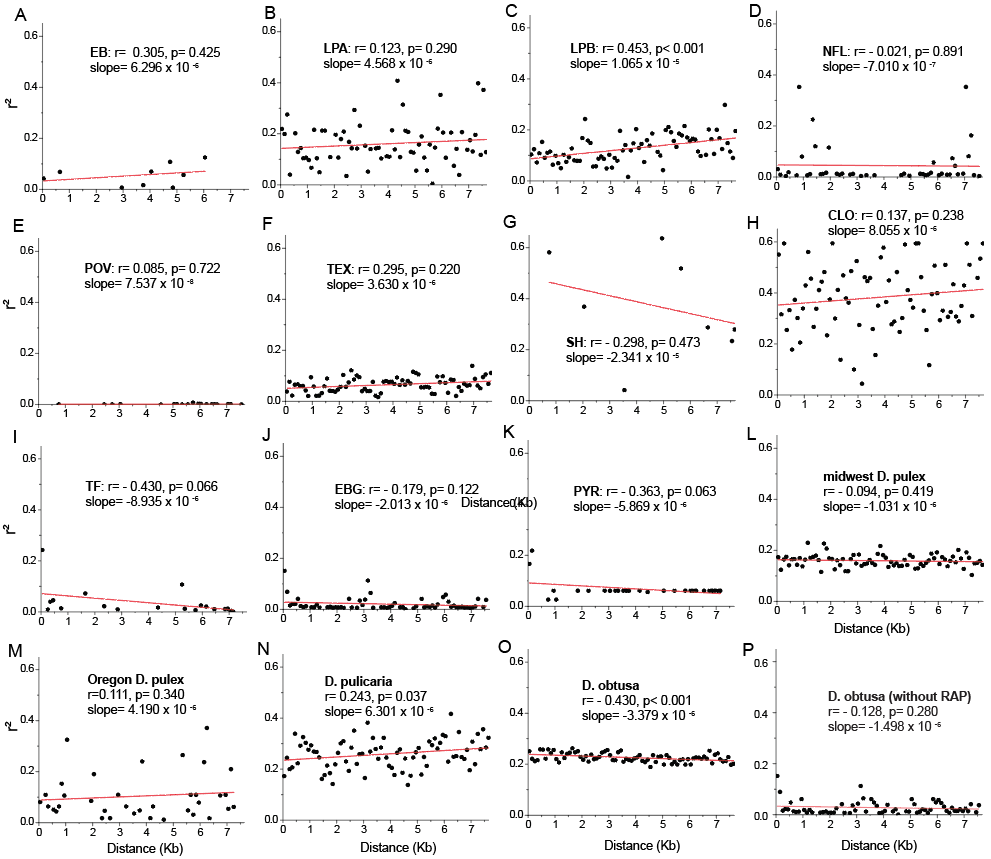


**Figure S5.** Neighbor-joining (NJ) tree for the *D. pulex*, *D. pulicaria*, and *D. obtusa* clones based on the full-length mitochondrial sequences. *D. magna* was used as an outgroup. Clones with two haplotypes are phased using allele frequencies, i.e., assigning all major alleles to one haplotype, and all minor alleles to the other. Haplotypes constructed from minor alleles are marked with solid circles and located at the tip of the corresponding branches. Arc **a)** shows the color-coded species, with species name listed on the left panel; Arc **b**) shows the color-coded populations; Arc **c**) indicates the density of the heteroplasmic sites within each clone. Stars indicate bootstrap values >75%. Inset on the bottom left shows the branch length of the major clades.


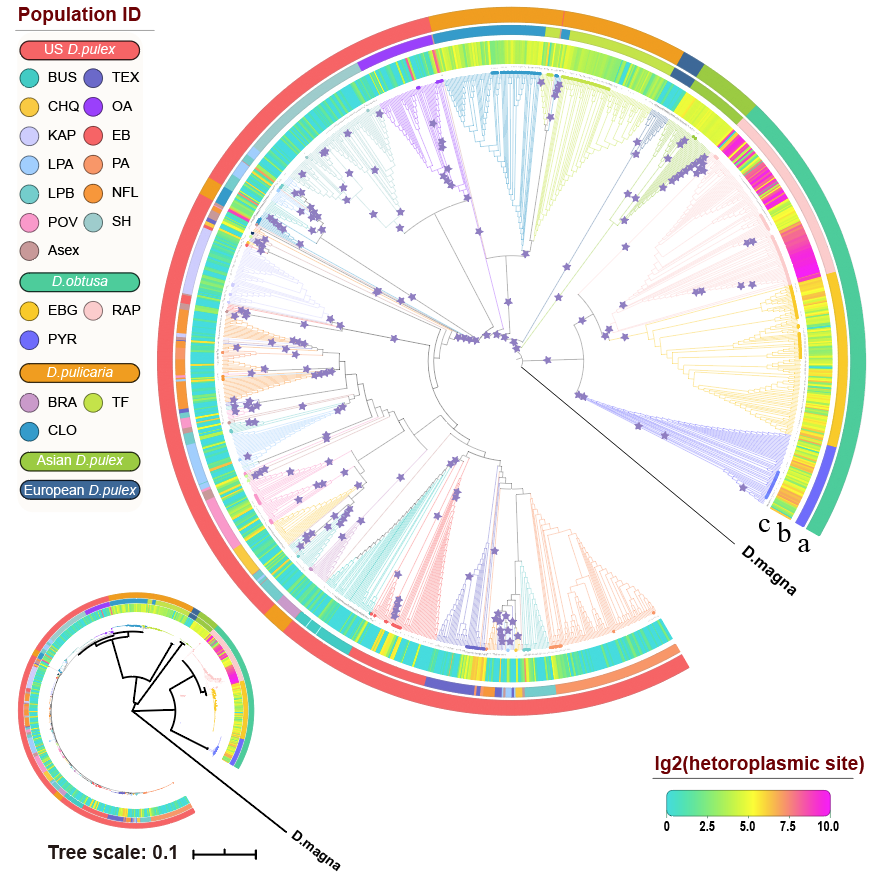


**Figure S6.** Mitochondrial phylogeny of the North American *D. obtusa* clones based on maximum-likelihood analysis of COⅠ gene sequences. Branch colors differentiate two haplotype clades identified in this study. Colored circles are *D. obtusa* samples from Penton et al. (2004), with blue and red representing the two clades designated in their analyses. Bootstrap values >75% are shown.


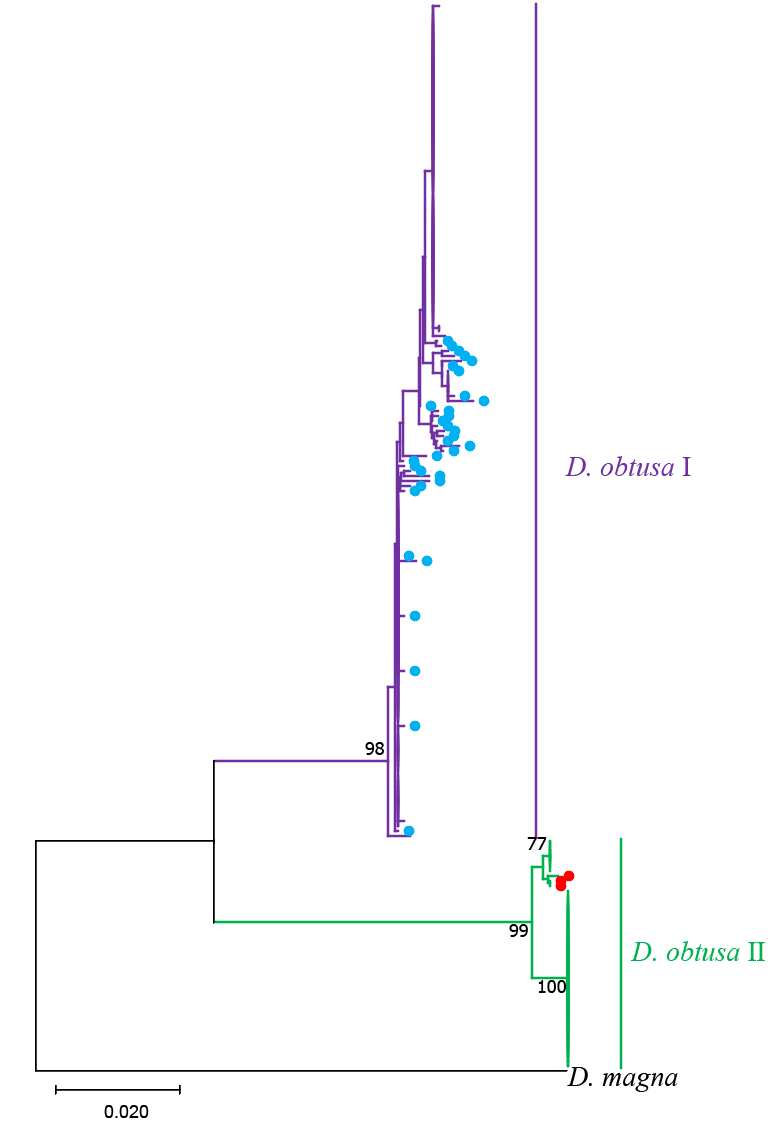


**Figure S7.** A) Association between F_st_ for the mitochondrial genome and geographic distance (Pearson r = 0.26; *p* = 0.08). B) Relationship between F_st_ for mitochondrial and nuclear genomes for the 10 North American *D. pulex* populations (Pearson r = 0.64; *p* <0.0001). Each dot is a pairwise comparison of two North American *D. pulex* populations.


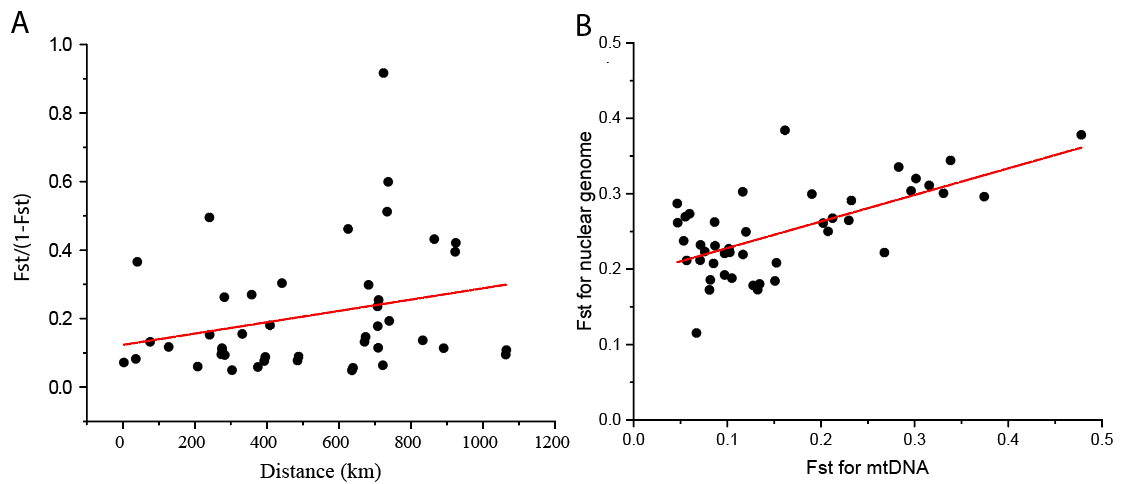


**Figure S8.** Median joining haplotype network for RAP population generated using PopART program (Leigh and Bryant 2015). Major and minor indicates haplotypes generated from major and minor alleles. Black circles are median vectors (hypothesized sequences to connect existing sequences within the network with maximum parsimony). Red bubble (top left) suggests that some major haplotypes could originate from existing minor haplotypes, likely involved recombination of the existing minor haplotypes. Green bubble (bottom left) suggests that minor haplotypes could involve from existing major haplotypes.


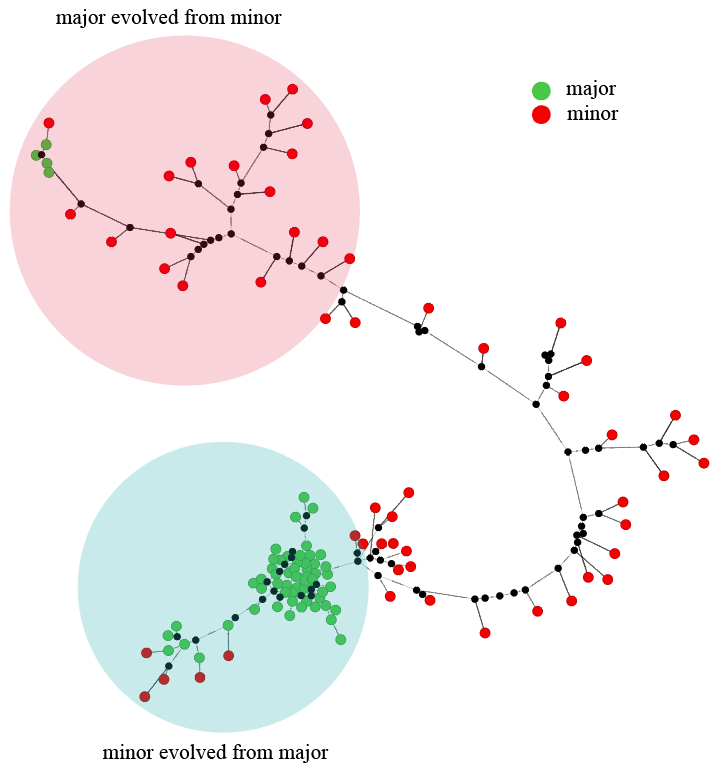


Leigh, JW, Bryant D. 2015. PopART: Full-feature software for haplotype network construction. *Methods Ecol Evol* 6(9):1110–1116.
